# Supplementary material for: The System’s Point of View Applied to Dielectrophoresis in Plate Capacitor and Pointed-versus-Pointed Electrode Chambers
Source: Micromachines (Basel). 2023 Mar 17;14(3):670. doi: 10.3390/mi14030670 (PMC10053818; doi:10.3390/mi14030670)
Supplement: Supplementary file 1 [file micromachines-14-00670-s001.zip › Figure S1.pdf]

Plots of the normal force components acting on the electrode walls

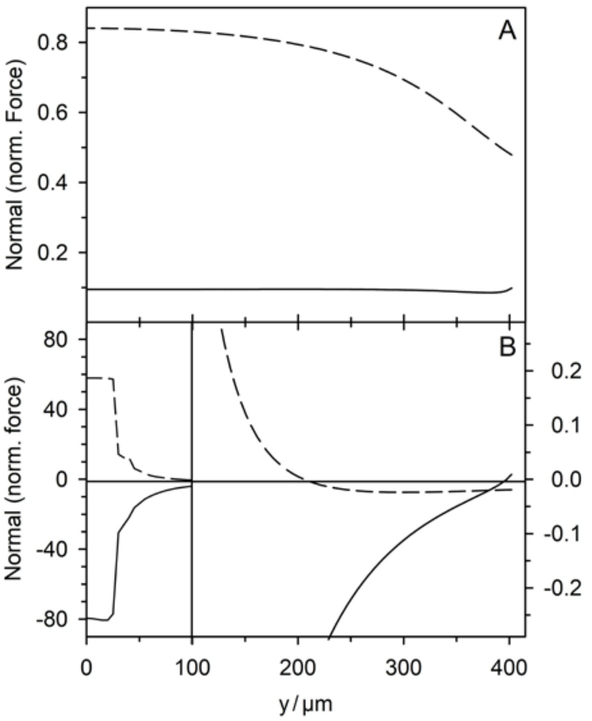

Figure S1: Normal force components acting on the left electrode of the plane-versus-plane electrode chamber (A) and on the left electrode wall of the pointed-versus-pointed electrode chamber (B). Long dashed lines: 1-S sphere in 0.1-S medium; solid lines: 0.1-S sphere in 1.0-S medium.

In each of the four cases (two chamber geometries, two complementary properties of spheres and media), the forces were calculated from the differences of the elements in the first two columns (U and V) of the conductance matrices in the Excel spreadsheets (Tables 1, 2, 3, and 4).

While only moderately high attractive forces are seen in the plane-versus-plane electrode chamber (A), strong repulsive forces and attractive forces act on the 0.1-S sphere and the 1.0-S sphere, respectively, near the electrode (left panel of B). The forces on both spheres decrease towards the edge of the chamber (right panel of B) and even change their signs.
